# Retracing Circulating Tumour Cells for Biomarker Characterization after Enumeration

**DOI:** 10.5772/60995

**Published:** 2015-06-30

**Authors:** Anders S. Frandsen, Anna Fabisiewicz, Agnieszka Jagiello-Gruszfeld, Anastasiya S. Haugaard, Louise Munkhaus Petersen, Katrine Brandt Albrektsen, Sarah Nejlund, Julie Smith, Henrik Stender, Thore Hillig, György Sölétormos

**Affiliations:** 1 CytoTrack ApS, Lyngby, Denmark; 2 Department of Translational and Molecular Oncology, The Maria Sklodowska-Curie Memorial Cancer Centre and Institute of Oncology, Warsaw, Poland; 3 Department of Breast Cancer and Reconstruction Surgery, The Maria Sklodowska-Curie Memorial Cancer Centre and Institute of Oncology, Warsaw, Poland; 4 CTC Center of Excellence, Department of Clinical Biochemistry, North Zealand Hospital, University of Copenhagen, Denmark; 5 Department of Technology, Faculty of Health and Technology, Metropolitan University College, Copenhagen, Denmark; 6 Department of Clinical Biochemistry, North Zealand Hospital, University of Copenhagen, Denmark

**Keywords:** CytoDisc, Ciculating tumor cells, CytoTrack, Cancer, HER2, CTC, Characterization, Enumeration, Breast cancer, Immunofluorescence, CK, Liquid biopsy, Method, Retracing, Metastasis

## Abstract

**Background:**

Retracing and biomarker characterization of individual circulating tumour cells (CTCs) may potentially contribute to personalized metastatic cancer therapy. This is relevant when a biopsy of the metastasis is complicated or impossible to acquire.

**Methods:**

A novel disc format was used to map and retrace individual CTCs from breast-cancer patients and nucleated cells from healthy blood donors using the CytoTrack platform. For proof of the retracing concept, CTC HER2 characterization by immunofluorescence was tested.

**Results:**

CTCs were detected and enumerated in three of four blood samples from breast-cancer patients and the locations of each individual CTCs were mapped on the discs. Nucleated cells were retraced on seven discs with 96.6%±8.5% recovery on five fields of view on each disc. Shifting of field of view for retracing was measured to 4-29 μm. In a blood sample from a HER2-positive breast-cancer patient, CTC enumeration and mapping was followed by HER2 characterization and retracing to demonstrate downstream immunofluorescence analysis of the CTC.

**Conclusion:**

Mapping and retracing of CTCs enables downstream analysis of individual CTCs for existing and future cancer genotypic and phenotypic biomarkers. Future studies will uncover this potential of the novel retracing technology.

## 1. Introduction

One of the hallmarks of cancer is tumour heterogeneity and genomic instability, which is a particular challenge in adjuvant treatment strategies [1,2]. Several biomarker-based tests for characterization of tumour tissue are used in clinical practice for directing treatments, and their number is rapidly increasing [3]. Today, these tests are predominantly performed on primary tumour tissue, although patients with relapse ultimately suffer from the direct sequelae of the metastatic lesions. Tumour-cell migration demands a transformation process (epithelial-mesenchymal transition), so phenotypical appearance may change compared to the characteristics of the primary tumour [4,5]. Furthermore, continuous genetic drift due to various sources – and subsequent natural selection in tumour cells overtime and under treatment – results in heterogeneity both within the primary tumour and in descendant metastatic lesions. The clinical importance of heterogeneity is evident both within primary tumours and between primary tumours and metastases [6]. Thus, there is a need for biochemical characterization of the metastatic lesion for tailor-made therapy; however, the inaccessibility of the metastatic sites is challenging, and taking a biopsy can be a cumbersome procedure for the patient. Analysing circulating tumour cells (CTCs) as as a surrogate for the metastatic lesion may improve treatment decisions and patient outcomes [7,8].

For more than a century it has been known that cancer cells detach from tumours and circulate in the blood stream, but only contemporary methods are capable of both detecting and enumerating the rare cells in the blood [9,10]. CTCs can repeatedly be isolated from peripheral blood of most cancer patients. Methods used in clinical practice are limited to enumeration of CTCs, where the number is predictive of outcome and is therefore of limited benefit for patients [11]. A blood sample containing CTCs is often referred to as a ‘liquid biopsy’ and may provide proxies of metastatic cancers [12,13]. The clinical significance of the intra- and intermetastatic heterogeneity still needs to be established. Like metastases, CTCs are probably heterogeneous populations of cells; however, they are of limited number and may not be able to reflect the heterogeneous nature of human cancers.

However, methods for characterization of CTC biomarkers are considered to have great promise for the practical implementation of personalized cancer treatment [7,13]. This may involve evaluation of the detected CTCs with, e.g., immunofluorescence or fluorescence in-situ hybridization (FISH) – always requiring retracing of the CTCs.

**Figure 1.**
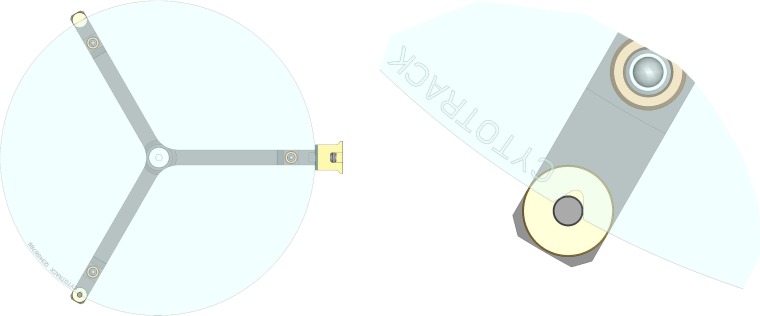
CytoDisc with ‘notch’ for repositioning. The area of the CytoDisc is 120 cm^2^, sufficient for a monolayer of more than 100 million nucleated blood cells.

CytoTrack™ is a novel scanning and imaging concept bridging flow cytometry and fluorescence microscopy [10]. From peripheral blood, all nucleated blood cells including CTCs are isolated and immobilized onto a round glass disc. In this study, a novel disc format called CytoDisc™ was assessed; alongside enumeration, the disc provides an accurate location of each CTC for potential downstream phenotypic or genotypic evaluation (Figure 1). The disc is tested with regard to the accuracy of mapping and retracing CTCs, and HER2 immunofluorescence is applied to test downstream biomarker characterization potential.

## 2. Materials and Methods

### 2.1 Clinical specimens

From metastatic breast cancer patients, samples of 7.5 mL peripheral blood were collected in CellSave Preservative Tubes (Veridex, Raritan, NJ, USA) The tube s were shipped and stored at ambient temperature and analysed within four days. The patients were from The Maria Sklodowska-Curie Memorial Cancer Centre and Institute of Oncology, Warsaw, Poland, and written informed consent was obtained from each participant prior to sample collection. The study was approved by the Ethics Committee of the Maria Sklodowska-Curie Memorial Cancer Centre and Institute of Oncology (No13/2008).

The 707528 patient was diagnosed with ER-pos, PgR-pos and HER2-neg (1+), Ki67>14% multifocal infiltrating ductal carcinoma, stage IIA. She had a mastectomy in 2009 and in 2012 a recurrence of carcinoma at the site of surgery. Reoperation was performed, followed by tamoxifen treatment and radiotherapy. In 2013, bone metastases were found, and the patient was treated with chemotherapy and palliative radiotherapy. The patient is now treated with denosumab.

The 1067856 patient was diagnosed in July 2014 with infiltrating ductal carcinoma, ER-10%, PgR-100%, HER2-neg (0), primary IIB stage with metastasis to bone, skin and lymph nodes, Ki67-10%. In September 2014 she was treated with letrozol and had bone radiotherapy. Now, the patient is treated with denosumab.

The 1033919 patient was diagnosed in 2013 with infiltrating ductal carcinoma, stage IIB, G3, ER-0%, PgR-0% and HER2-pos (3+), Ki67- 80%. The patient was treated with trastuzumab followed by a mastectomy in August 2014. In September 2014, metastasis to bone and liver, and recurrence in chest wall were found. The patient is now treated with lapatinib and capecitabine.

The 673451 patient was diagnosed in 2002 with invasive ductal carcinoma G3, pT2N0, ER-50%, PgR-0%, HER2- neg. A radical mastectomy in January 2003 was followed by adjuvant chemotherapy. She has metastasis to lung, bone, lymph node and soft tissue of the skull base. The patient was treated with palliative chemotherapy with docetaxel and cisplatin.

The 790864 patient was diagnosed with ER-neg, PgR-neg and HER2-pos (2+), ductal carcinoma, cT3NxM0 in 2007. Following four courses of AC chemotherapy she underwent a radical mastectomy, and, because of lymph-node involvement, she was treated with adjuvant therapy and trastuzumab. In 2014 metastasis to bone and bone marrow was found, and the patient was treated with paxitaxel and denosumab. In April 2015 liver progression was diagnosed, and change of treatment is planned.

### 2.2 Donor Specimens

Peripheral blood samples from healthy donors (Nordsjællands Hospital, Hillerød, Denmark) were collected in EDTA-tubes and analysed within four hours. Written informed consent was obtained from each donor.

### 2.3 CTC enumeration

**Figure 2.**
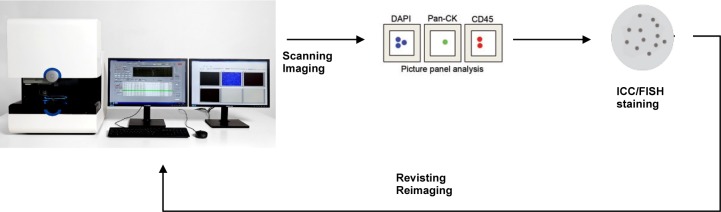
The CytoTrack system is a scanning and imaging technology bridging flow cytometry and fluorescence microscopy. After detection and enumeration of CTCs, each individual CTC can be further analysed for instance for immunofluorescence (e.g., HER2 antibody). By reinserting the CytoDisc in the CytoTrack system, CTCs can be retraced and characterized by imaging.

CTCs were enumerated according to the manufacturer's instructions (Figure 2). Briefly, blood samples were centrifuged at 2500 g for 15 minutes and the buffy-coat layer with nucleated cells and possible CTCs were transferred to 15 mL tubes. Remaining red blood cells were lysed and nucleated cells were fixed with FACS Lysing solution (BD Biosciences) for 15 minutes and centrifuged at 2500 g for 15 minutes. CTCs were stained using CTC Stain (CytoTrack ApS, Lyngby, Denmark) comprising a mixture of anti-CD45/NIR antibody, anti-EpCAM/Yellow and anti-cytokeratin/Green antibody and nuclear-stain DAPI for one hour at 2-8°C in darkness. Subsequently, the cells were washed with PBS with 1% BSA and re-suspended in 1 mL H_2_O. The cell suspension was smeared onto the CytoDisc, air-dried, mounted using mounting medium (Olink Bioscience, Uppsala, Sweden), covered with a CytoCover™ (CytoTrack) and sealed with rubber cement (FixoGum, Marabu Scandinavia, Denmark) along the edge. The CytoDisc was inserted into a CytoTrack CT4 platform (CytoTrack) and locked into position using the ‘notch’ on the CytoDisc (Figure 1) and focused using the five pre-set focus points. Subsequently, CTCs were detected and enumerated by scanning using the Green channel with a 20x scanning objective. Scanning was performed within one week of staining. Green events were recorded and listed in a hotspot table. Recorded events were visually inspected by the operator in the Green channel and an image gallery was automatically generated using DAPI, Green and NIR channels and 20x imaging objective from positions on the CytoDisc with possible CTCs. The image gallery was analysed using the following morphologic criteria: nearly round and size >4 μm, with visible DAPI-positive nucleus with at least 50% association with the cytoplasm, cytokeratin-positive, and CD45-negative. The definition of CTC is similar to the definition used by other CTC analysis methods [14][Bibr bibr15-60995][Bibr bibr16-60995][Bibr bibr17-60995]–[18].

### 2.4 CTC Mapping

A map of positions of individual CTCs on the CytoDisc was constructed after scanning the blood samples from breast-cancer patients.

### 2.5 Retracing of cells on CytoDisc

The FixoGum seal was removed and the CytoDisc with CytoCover was immersed in PBS in a 145-mm-diameter Petri dish. After approximately 15 minutes the CytoDisc was carefully lifted into a vertical position allowing the CytoCover to gently slide off, and was carefully washed with PBS to remove remaining mounting medium. The CytoDisc was air dried and subsequently stored refrigerated. The CytoDisc was then inserted into the CytoTrack scanner and locked into position using the ‘notch’ (Figure 1). Five fields of view on the five focus points for the CytoDisc were captured and the number of cells was counted in each field of view using ImageJ before and after removal of CytoCover and mounting medium. Cell recovery was investigated by retracing cells in five fields of view using ImageJ (version 1.48v), an open-source image-processing program for cell counting. The cell counts were used for estimation of the recovery, i.e., all cells were counted on selected fields of view on the CytoDisc and the same fields of view were retrieved and counted again. First, images were turned into greyscale and the threshold was adjusted to highlight all the objects to be counted. To eliminate noise, the background was subtracted with a rolling ball with a radius of 50 pixels. The ‘Watershed’ function was applied in cases where objects were merged together. The function ‘Analyse particles' was used to count objects. Only particles with pixel size above 300 and circularity within the range of 0-1.0 were encountered. If the cells had shifted position at the second count (recovery), the shifted distance was measured using ImageJ.

The following variations to the manufacturer's standard procedure (see *CTC enumeration* under *Materials and Methods)* were tested to see whether they had any effect on retracing of the blood cells: 1. Drying the cell smear on the CytoDisc at 37°C instead of room temperature. 2. For re-suspension of the cell pellet, 0.5 mL and 2 mL H_2_O was used instead of the standard 1 mL H_2_O. 3. During the cell-smear drying process at room temperature, the CytoDisc was continuously rotated automatically to test if this improved the cell distribution for detection, enumeration and retracing. 4. Storage of the CytoDisc for up to four weeks at 2-8°C was also studied. The CytoDisc was scanned and rescanning/retracing was performed three and four weeks later.

### 2.6 HER2 Characterization

After enumeration, one patient sample (ID790864) was HER2-stained and the CTCs were retraced for characterization. On the CytoDisc with the enumerated CTCs, 400 μl of HER2/NIR antibody (clone 24D2, Biolegend, San Diego, CA) diluted 1:200 in PBS were applied, and a CytoCover was put on top to ensure uniform coverage of the disc surface. The CytoDisc was incubated for 1 hr at room temperature in a closed Petri dish. After incubation, the CytoCover was removed and the CytoDisc washed with PBS as described above. After air-drying, mounting medium and CytoCover were applied.

## 3. Results

### 3.1 CTC enumeration and mapping

**Figure 3.**
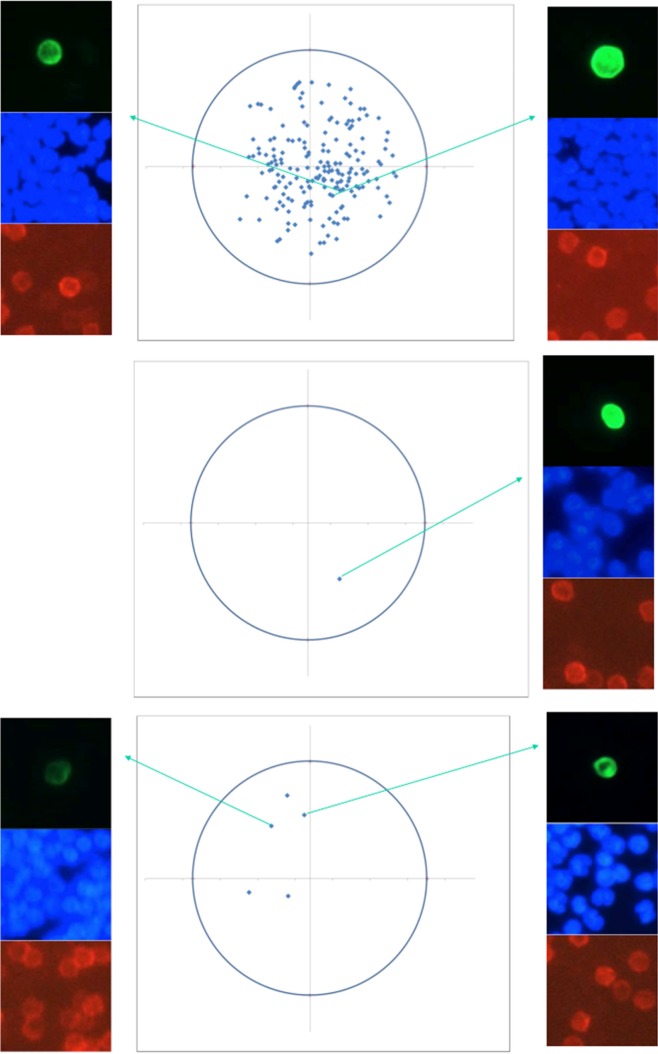
Representative examples of CTCs from three breast-cancer patients (ID 707528, 1067856 and 673451) with 189, one and five CTCs, respectively. Images (anti-cytokeratin/Green, nuclear stain DAPI/Blue, anti-CD45/NIR) confirm the presence of individual CTCs at the locations on the map.

Enumeration of CTCs was conducted in four specimens from four metastatic breast cancer patients. CTCs were detected in three of the samples and enumerated at 189, one and five, respectively. The presence of individual CTCs at all map positions was confirmed by imaging. Representative results of CTCs linking images of individual CTCs to the map locations are shown in Figure 3 for each of the three specimens.

### 3.2 Cell recovery and precision

The cell recovery of the retracing procedure using seven blood-donor samples was analysed by the standard procedure for CTC enumeration. For one sample, recovery was measured three times over a one-month period. In addition, variations to the standard procedure were also investigated. The results are summarized in Table 1.

**Table 1.** Recovery for retracing of nucleated cells

			Within* CytoDisc		Total CytoDisc	
				
Disc (#)	CTC Enumeration Procedure	Retracing (time)	Recovery	CV%	Recovery	CV%
1	Standard^1^	Same day	97%	2.3%		
2	Standard	Same day	98%	4.5%		
3	Standard	Same day	98%	6.6%		
4	Standard	Same day	95%	10.3%	96.6%	8.5%
5	Standard	Same day	93%	17.0%		
6	Standard	Same day	98%	10.7%		
7	Standard	Same day	97%	8.1%		
		
7	Standard	Same day	97%	8.1%		
7	Standard	3 weeks	96%	19.3%	96.0%	13.8%
7	Standard	4 weeks	96%	14.1%		
		
8	Drying 37°C^2^	Same day	97%	2.3%		
8	Drying 37°C	3 weeks	104%	7.3%	100%	4.6%
8	Drying 37°C	4 weeks	99%	4.2%		
		
9	0.5 mL H_2_O^3^	Same day	104%	10.3%		
9	0.5 mL H_2_O	3 weeks	111%	11.2%	109%	10.3%
9	0.5 mL H_2_O	4 weeks	111%	9.3%		

10	2 ml H_2_O^4^	Same day	99%	3.6%		

11	Rotating^5^	Same day	97%	7.0%		

1 CTC enumeration according to manufacturer's instructions

2 CTC enumeration according to ^1^, but modified by drying cell smear on CytoDisc at 37°C.

3 CTC enumeration according to ^1^, but modified by use of 0.5 mL H_2_O for cell pellet re-suspension.

4 CTC enumeration according to ^1^, but modified by use of 2 mL H_2_O for cell pellet re-suspension.

5 CTC enumeration according to ^1^, but modified by an automated continuous rotation of the disc during drying of cell smear at room temperature.

* Five preset focus points (fields of view) on the CytoDisc

Overall recovery for the standard procedure was 97%. None of the modifications to the standard procedure, i.e., higher and lower amount of H_2_O for pellet re-suspension, heating to 37°C during drying, and rotating of the CytoDisc, showed an impact on cell recovery.

**Figure 4.**
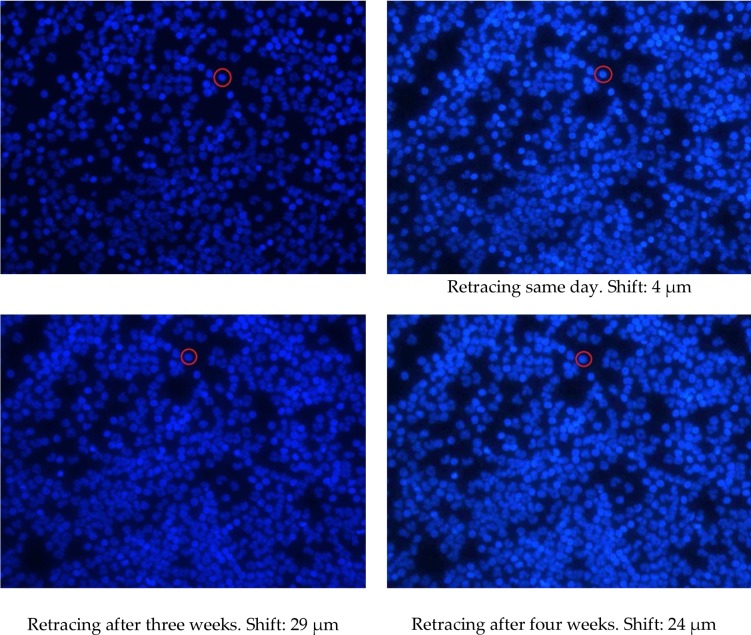
Same field of view before and after removal of CytoCover and mounting medium. After removal, the disc was imaged on the same day and after three and four weeks to measure potential distance shift in fields of view. Levels of precision are slightly different between image acquisitions. The image position shift is illustrated by the red circle around a cell.

**Figure 5.**
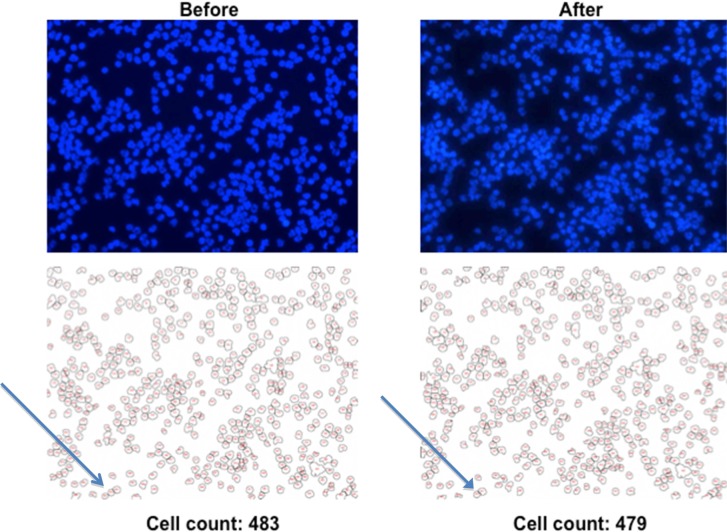
Images (top) and cell count (bottom) of nucleated cells before and after removal of CytoCover and mounting medium. The arrows illustrate examples of differences in cell counting caused by shifting of the field of view. Thus, some cells may be visible in one field of view but not the other.

Images of one field of view for the CytoDisc where retracing was performed three times are shown in Figure 4. Shifting of field of view position is illustrated by the shift of a red circle around one individual cell. The actual shifting distance was 4, 29 and 24 μm, respectively, relatively small compared to the size of the field of view of 510 × 384 μm. Cell counting is illustrated in Figure 5 and shows that very few cells are lost in the process and most of the differences before and after retracing are due to counting errors for clusters of cells and cells on the edges. The recovery may therefore be even closer to 100% than reported in Table 1.

### 3.3 HER2 characterization of individual CTCs

**Figure 6.**
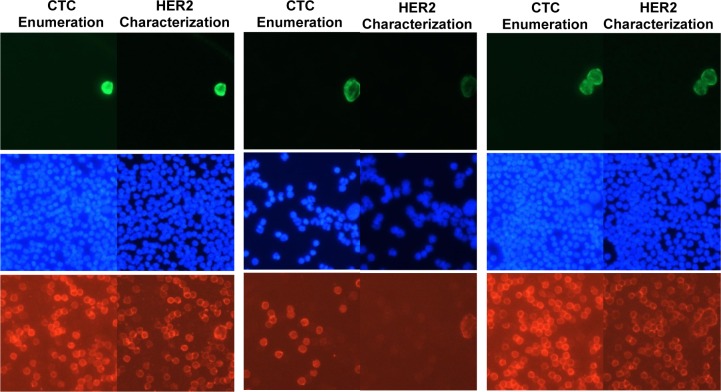
Images (anti-cytokeratin/Green, nuclear stain DAPI/Blue, anti-HER2/NIR and/or anti-CD45/NIR) of the same fields of view after CTC enumeration (left) and after HER2 characterization (right) for three fields of view containing four CTCs. HER2 membrane staining of each individual CTC is observed together with CD45 membrane staining after HER2 characterization.

A blood sample from a patient with HER2-positive primary tumour (ID 790864) was used to exemplify the process of CTC enumeration followed by HER2 characterization of the individual CTCs. The same channel/dye fluorescence was used for both CD45 (negative control for CTC enumeration) and HER2 characterization. This way HER2 characterization could be observed as NIR staining of the CTC after staining with HER2/NIR antibody. A total of four CTCs were enumerated and the positions mapped. After removal of CytoCover and mounting medium, HER2 characterization was performed and the individual CTCs were all retraced and shown to be HER2-positive, as illustrated in Figure 6.

## 4. Discussion

CTCs may serve as a liquid biopsy in metastatic cancer, and this must involve CTC characterization beyond detection and enumeration. Thus, CTCs need to be retraced for further analysis. We found that retracing of blood cells on the CytoDisc is possible after up to four weeks of storage of the disc. As proof of the concept, CTCs from a breast-cancer patient were enumerated and subsequently retraced and characterized with HER2 antibody. To our knowledge, this is the first study to analyse breast-cancer patient specimens using the CytoTrack system. From the patient cells on the disc, we generated a map of the CTCs which allows retracing of individual CTCs for subsequent biomarker analysis.

Treatment of metastatic breast cancer is currently guided by characterization of cells of the primary tumour, although 90% of deaths due to breast cancer occur as a consequence of metastases. In up to 50% of all breast cancer patients the molecular profile of the metastases is different from the primary tumour that is currently guiding treatment [8,19]. Interest in investigating whether CTCs can be used as a tool for characterization of the metastatic events has therefore begun to grow [7]. Whole-cell analysis methods often require selection or enrichment of CTCs by morphological properties, size or selection/depletion by immunocapture techniques [14]. The CytoTrack system immobilizes all nucleated cells without prior selection, depletion or enrichment and furthermore provides a combined concept for both enumeration and characterization [10].

Beside CytoTrack other methods are looking beyond enumeration, such as EPIC Science's CTC platform or the FDA-approved CellSearch method – the latter in combination with for example the DEPArray system – to uncover mutations of individual PTCs [19]. As the enumeration procedures are linked to epithelial biomarkers, such as cytokeratins in CytoTrack and the HD-CTC assay, or EpCAM in CelSearch, current methods have advantages and pitfalls [20,21]. During the CTC detection/enumeration step some cancer cells in a blood sample might not be traced with the specific biomarkers applied by the assays because of the possibly heterogenic population of CTCs, where mutations and epithelial-mesenchymal transition may have occurred. Only larger clinical studies can reveal whether present CTC enumeration and characterization metods can unravel the individual metastatic heterogeneity to a degree that will refine treatment strategies and achieve better outcomes.

In this study, we confirmed the applicability of the CytoDisc for retracing of CTCs, and studies are ongoing in our group to develop downstream analysis of existing cancer biomarkers, such as HER2 in breast cancer. The HER2 characterization of individual CTCs and retracing in one patient in this study provides proof-of concept for such on-disc downstream analysis. Future studies must investigate if the CytoTrack method is compatible for further downstream analyses such as, e.g., FISH, and rolling circle amplifications (RCA) using different types of markers for proteins, RNA and DNA.

The generated map facilitates individual CTC imaging both during the initial CTC enumeration and during the subsequent characterization. The map may also serve as a template for application of reagents directly onto individual CTCs, thus limiting the volume of reagents required for analysis.

This study shows that storage of the disc is possible and cells can be retraced after four weeks of storage. However, we have not shown if storage is compatible with specific CTC characterization analysis. This certainly also depends on the stability of the chosen biomarker at different temperatures, and possibly other conditions. Potentially, individual CTCs may even be analysed multiple times provided the tested biomarkers are stable. Thus, storage potential is a relevant aspect for future studies on specific biomarkers.

The CytoTrack system with the new CytoDisc allows retracing and potentially extended CTC characterization; future studies may use this to test specific CTC biomarker analyses. CTC characterization has the potential to enhance personalized metastatic cancer therapy.

## 5. Compliance with ethical research standards

All research on human subjects presented in this paper was conducted in accordance with the ethical research standards prescribed by the responsible national/institutional committee on human experimentation and with the WMA Declaration of Helsinki as of its seventh revision in 2013. Informed consent was obtained from all human subjects participating in the study.

Anders S. Frandsen, Anastasiya S. Haugaard, Louise Munkhaus Petersen, Katrine Brandt Albrektsen, Sarah Nejlund and Henrik Stender were employed by CytoTrack ApS during the development and documentation of the retracing feature. Additional authors declare no conflict of interest.
